# Efficacy of oral tapentadol immediate-release for the management of moderate-to-severe cancer pain in patients presenting to the oncology ward of a tertiary care hospital

**DOI:** 10.12669/pjms.42.1.12885

**Published:** 2026-01

**Authors:** Saima Zahoor, Ammara Manzoor, Hareem Akhtar, Sana Nasir, Hina Gul

**Affiliations:** 1 Saima Zahoor, DMRT, CIMP; Fellowship in Palliative Care, Department of Medical Oncology, Jinnah Postgraduate Medical Centre, Karachi, Pakistan; 2 Ammara Manzoor, FCPS Oncology Department, National Institute of Blood Diseases, Karachi, Pakistan; 3 Hareem Akhtar, MBBS, Department of Medical Oncology, Jinnah Postgraduate Medical Centre, Karachi, Pakistan; 4 Sana Nasir, MBBS, Department of Medical Oncology, Jinnah Postgraduate Medical Centre, Karachi, Pakistan; 5 Hina Gul, MBBS, Department of Medical Oncology, Jinnah Postgraduate Medical Centre, Karachi, Pakistan

**Keywords:** Analgesic agents, Cancer pain, Chronic pain, Oncological pain, Tapentadol

## Abstract

**Objectives:**

To assess the efficacy and determine an effective dosing range of oral tapentadol immediate-release (IR) for managing chronic, moderate-to-severe pain in cancer patients in Pakistan, where extended-release formulations are not readily available.

**Methodology:**

This dose-response study was conducted at the Department of Medical Oncology, JPMC, Karachi over a one-week period. Forty-five patients were given 75 mg BID of oral tapentadol IR and the medication was titrated as necessary to achieve optimum analgesic effects. Pain before and after one week of commencing the medication was assessed by an 11-point Numeric Rating Scale. Patient satisfaction, adverse effects and dosage required to achieve adequate analgesia was noted. Data was analyzed using IBM SPSS version 23.

**Results:**

There was a significant decrease in the NRS score (*p* <0.005), from a mean score of 7.86 (SD 1.71) at the commencement of the study period to 3.14 (SD 2.86) after one week of oral tapentadol therapy. Although the dosage increased significantly over the study period, the extent of pain relief did not differ significantly across the different dosage groups (*p* =0.23). A majority of patients (65%; *n*=23) reported no adverse effects. No relationship between the level of pain reduction and gender (*p* =0.28), cancer type (*p* =0.68), or previous analgesic use (p =0.096) were found.

**Conclusion:**

A starting dose of 75 mg BID of tapentadol IR led to a significant pain reduction in cancer patients with chronic pain in the Pakistani population, regardless of previous analgesic intake or cancer type.

## INTRODUCTION

Pain is a major feature of cancer, with 44% of all cancer patients having experienced some form of pain.^1^ Adequate management of cancer pain is a challenging domain, given its severity, chronic nature and mixed nociceptive-neuropathic characteristics.^2^ The inadequate treatment of oncological pain is a well-documented phenomenon, particularly in low-income countries. This can be attributed to delay in the diagnosis and treatment of cancer and inadequate pain assessment in cancer patients.^3^ The WHO analgesic ladder recommends a simple approach to pain management, by stepwise administration of more potent analgesics according to the severity of the pain.^4^

Tapentadol is a centrally acting analgesic with a unique dual-mechanism of action, acting both as a μ-opioid receptor agonist and a noradrenaline reuptake inhibitor.^5^ ’Tapentadol has been shown to have an analgesic effect comparative to conventional opioid agonists in treating cancer pain, with the added benefits of better tolerance and a lower frequency of adverse effects, which can be attributed to its lower affinity to the μ-opioid receptor and less serotonergic activity. This synergistic mechanism of action might play a role in alleviating the mixed nociceptive-neuropathic nature of cancer pain.^5^

While there is no central cancer registry in Pakistan, the available data shows a high prevalence of cancer in the country.^6^ Furthermore, most cancer patients present at advanced stages, making analgesic control more difficult, since cancer pain has been shown to increase with disease progression.^7^ Most cancer patients only survive five or more years after diagnosis, which highlights the need for adequate analgesic regimens as part of palliative treatment.^8^ There is also a dearth of literature regarding cancer pain management in the Pakistani population, but available data shows that the rate of undertreatment of cancer pain is significantly higher than the global average.^9^

Tapentadol, with its relatively better tolerability even at higher doses, is a potentially suitable alternative to other, traditional analgesic agents. While multiple studies have assessed the efficacy of tapentadol ER (extended-release) or PR (prolonged-release) for managing chronic cancer pain, none have been conducted on the effectiveness of tapentadol IR (immediate-release) for the given indication. Since the ER/PR formulation is currently not available in Pakistan, this study aimed to assess the effectiveness of oral tapentadol IR and establish an appropriate dosing range for managing moderate-to-severe cancer pain.

## METHODOLOGY

This dose-response study was conducted at the Department of Medical Oncology, Jinnah Postgraduate Medical Center, Karachi, over a period of one week. By considering a medium effect size of 0.5^10^, α=0.05, a power of 80% and an attrition rate of 0.7 based on previous studies,^11^ a sample size of 45 was calculated. A non-probability, convenient sampling technique was used to select the eligible participants. The inclusion criteria were adult cancer patients of either gender having chronic, moderate-to-severe pain, which was classified as pain over four in an 11 points Numeric Rating Scale (from 0 to 10).^12^

### Ethical Approval:

It was obtained from the institutional IRB Ref.l (No.F.2-81/2024-GENL/265/JPMC; dated: May 20, 2024).

Informed consent was obtained from all participants prior to participation. Demographic data and history of analgesic intake was recorded on a structured proforma. After discontinuing all previous analgesic medications, patients were initiated on 75 mg BID of oral tapentadol IR (Tanpado ®) and the medication was titrated across three categories, namely 75mg BID, TID and QID, after every 48 hours as necessary to achieve optimum analgesic effects. Pain before and after one week of commencing therapy was assessed by an 11 points Numeric Rating Scale. The single-arm exploratory design,^13^ one-week follow-up point^14^, and sample size^11^ are consistent with previous studies assessing the initial efficacy of tapentadol ER for chronic pain management. Patient satisfaction and ease of usage were assessed via four points Likert scales (poor, fair, good and excellent). Effective dosage and adverse effects were noted on follow-up.

Data was analysed by using IBM SPSS version 23 with a 95% confidence interval, and a p-value of less than 0.05 was considered statistically significant. Frequency distribution for categorical factors was calculated and effect modifiers like gender, cancer type and previous analgesic therapy were compared. The difference in pain was assessed with the Wilcoxon signed-rank test. The Kruskal-Wallis test was used to assess the difference in dosage after one week.

## RESULTS

Of the 45 patients initially enrolled in the study, 35 completed the one-week course of tapentadol. Two patients expired during the study period, four patients were lost to follow-up and four patients discontinued the medication due to non-compliance. The mean age of participants was 49, with 60% being female and 40% male. The top three cancer types among the participants were breast (31.4%; *n*=11), lung (17.1%; *n*=6) and gastric (11.4%; *n*=4), respectively. Most patients had stage three cancer (57.13%; *n*=20), followed by stage four (33.3%; *n*=12), and two (8.6%; *n*=3). The most common comorbids were hypertension (40%; *n*=14), diabetes (37.1%; *n*=13) and arthritis (17.1%; *n*=6)respectively.

All study participants had a positive history of previous analgesic intake. Eleven patients had been using oral tramadol and three were using the injectable form. Seven patients had been prescribed NSAIDs, whereas three had a history of paracetamol therapy and one patient had been using pregabalin.

Patients had a mean NRS score of 7.86 (SD 1.71) prior to tapentadol commencement, which decreased significantly (*p*<0.005) over the study period to a mean of 3.14 (SD 2.86). The dosage of tapentadol increased significantly over the study period (*p*<0.0005). While 19 patients did not escalate the initial 75 mg BID dose, 25 patients increased the dosage to 75 mg TID and four patients to 75mg QID. The mean final dose was 192.9mg. There were no significant differences between the different dosage groups and the level of pain reduction (*p* =0.23) (Table-I).

**Table-I:** Difference in pain before and after one week of oral tapentadol IR therapy.

Effective Dose		Pain before tapentadol therapy	Pain after 1 week of tapentadol IR therapy	Difference in Pain
75mg BID	Mean	7.05	2.32	4.7368
	*n*	19	19	19
	Std. Deviation	1.649	2.473	2.76570
75mg TID	Mean	8.83	3.50	5.3333
	*n*	12	12	12
	Std. Deviation	1.403	3.060	2.90245
75mg QID	Mean	8.75	6.00	2.7500
	*n*	4	4	4
	Std. Deviation	.957	2.449	3.20156
Total	Mean	7.86	3.14	4.7143
	*n*	35	35	35
	Std. Deviation	1.717	2.861	2.87557

Patients were asked about common opioid related adverse effects on a checklist at follow-up as well as any other adverse effects they experienced. Most patients (65%; *n*=23) reported no adverse effects. The most common adverse effects were sedation (22%), followed by dizziness (17.2%) and nausea (8.6%). No relationship was found between the level of pain reduction and gender (*p* =0.28), cancer type (*p* =0.68), cancer stage (*p* =0.1), or previous analgesic use (*p* =0.096), or between the effective dosage and the type of cancer (*p* =0.423). Most participants reported excellent levels of satisfaction (57.1%) and ease of usage (94.3%), which were assessed via 4-point Likert scales.

## DISCUSSION

Our findings show that oral tapentadol IR at a starting dose of 75 mg BID is an appropriate analgesic therapy for managing chronic, moderate-to-severe cancer pain in the Pakistani population. This is similar to findings of other studies conducted on tapentadol ER, which is generally preferred for chronic pain management due to its longer duration of action, requiring less frequent dosage.^11^,^13^,^15^

Similar to other studies,^15^ our findings suggest that tapentadol can be a suitable option for opioid-naïve individuals as well as those who have a history of less potent opioid usage. Few studies have compared the efficacy of tapentadol therapy in relieving pain across different cancer types and stages, each of which can present with distinct pain phenotypes.^13^ Similar to available data on tapentadol ER, our study showed that the difference in pain relief and effective dosage of tapentadol IR was not related to cancer type. The low dropout rate (8.89%) in the present study is potentially attributable to the reduced incidence of opioid-related side effects. The frequency of adverse effects in our population is comparable to or lower than in other studies conducted on tapentadol ER, which, in turn, showed a lower incidence of opioid-related side effects compared to other, more potent opioid analgesics.^16^-^18^

Studies suggest that a significant proportion of cancer patients in Pakistan do not receive sufficient pain management, with one single-centre study showing that only a third of patients with advanced cancer received adequate analgesia.^9^ The high rates of untreated cancer pain are reflective of a general lack of palliative care services in the country. Inadequate public healthcare infrastructure, stringent government regulations regarding drug importation, and lack of palliative care services contribute to ineffective pain management.^19^ Morphine, a potent analgesic generally used for alleviating cancer pain, is a controlled drug in the country, and requires approval from four different governmental departments for procurement.^20^ These barriers in accessing effective pain management contribute to the high rates of untreated cancer pain in the country. The present study is the first to assess the efficacy of tapentadol IR in managing chronic cancer pain, across different cancer types and stages, and serves to highlight the potential of tapentadol IR as a practical alternative in settings with limited access to other pain medications. The low dropout rate and incidence of adverse effects are also findings which support the use of tapentadol IR therapy as compared to traditional opioids. We hope that this study informs future, large-scale studies into the long-term efficacy and comparative effectiveness of tapentadol IR in cancer patients.

### Limitations

The current study has several limitations, including the single-center nature, limited sample size, single follow-up point, and lack of a control group. Furthermore, the lack of significant differences in pain reduction between the different dosages groups may be attributable to insufficient power for assessing a dose-response relationship.

## CONCLUSION

A starting dose of 75 mg BID of oral tapentadol IR is an appropriate analgesic therapy for patients with chronic cancer pain in Pakistan, who suffer from high rates of untreated pain due to unavailability of potent opioid agonists and tapentadol ER/PR.

### Authors’ Contribution:

**SZ, SN and AM:** Conceived and designed the study protocol.

**HA and HG:** Did data collection and statistical analysis.

**AM and HA:** Drafted and edited the manuscript.

All authors read and approved the final version of the manuscript and vouch for the integrity of the study.
